# The Sanitary Era, or Progressive Health Journal

**Published:** 1891-08

**Authors:** 


THE SANITARY ERA, OR PROGRESSIVE HEALTH JOURNAL. William C. Conant, Publisher, P. O. Box 3059, New York City. Subscription price, $1.00 a year. Single copies, 10 cents.

The May number of this journal is at hand. It is a most excellent publication, intended not alone for the sanitarian but for citizens, mothers, nurses, invalids—everybody. The present number has a department devoted to water purifying, another to sanitary subjects in general, and another to protective hygiene.

